# Factors associated with the decline in under five diarrhea mortality in Tanzania from 1980-2015

**DOI:** 10.7189/jogh.09.020806

**Published:** 2019-12

**Authors:** Honorati Masanja, Pyande Mongi, Jitihada Baraka, Bianca Jackson, Yasinta Kisisiwe, Karim Manji, Nemes Iriya, Theopista John, Said Kimatta, Neff Walker, Robert E Black

**Affiliations:** 1 Ifakara Health Institute, Dar-es-Salaam, Tanzania; 2 Ministry of Health Community Development Gender Elderly and Children, Dodoma, Tanzania; 3 Muhimbili University of Health and Allied Sciences, Dar-es-Salaam, Tanzania; 4 Management Sciences for Health, Dar-es-Salaam, Tanzania; 5 World Health Organization, Dar-es-Salaam, Tanzania; 6 Johns Hopkins University, Bloomberg School of Public Health, Department of International Health, Institute for International Programs, Baltimore, Maryland, USA

## Abstract

**Background:**

Tanzania has made great progress in reducing diarrhea mortality in under- five children. We examined factors associated with the decline and projected the impact of scaling up interventions or reducing risk factors on diarrhea deaths.

**Methods:**

We reviewed economic, health, and diarrhea-related policies, reports and programs implemented during 1980 to 2015. We used the Lives Saved Tool to determine the percentage reduction in diarrhea-specific mortality attributable to changes in coverage of the interventions and risk factors, including direct diarrhea-related interventions, nutrition, and water, sanitation and hygiene (WASH). We projected the number of diarrhea deaths that could be prevented in 2030, assuming near universal coverage of different intervention packages.

**Results:**

Diarrhea-specific mortality among under-five children in Tanzania declined by 89% from 35.3 deaths per 1000 live births in 1980 to 3.9 deaths per 1000 live births in 2015. Factors associated with diarrhea-specific under-five mortality reduction included oral rehydration solution (ORS) use, changes in stunting prevalence, vitamin A supplementation, rotavirus vaccine, change in wasting prevalence and change in age-appropriate breastfeeding practices. Universal coverage of direct diarrhea, nutrition and WASH interventions has the potential reduce the diarrhea-specific mortality rate by 90%.

**Conclusions:**

Scaling up of a few key childhood interventions such as ORS and nutrition, and reducing the prevalence of stunting would address the remaining diarrhea-specific under-five mortality by 2030.

Tanzania is one of the 20 countdown priority countries that achieved Millennium Development Goal (MDG) 4 in 2015 of reducing child mortality by two thirds from the 1990 baseline [1]. Under five mortality declined from 161 deaths per 1000 livebirths to 54 deaths per 1000 live births [2].

In spite of the significant reductions in child deaths, diarrhea is still an important cause of child death in Tanzania [3]. The scale up of interventions and reduction of prevalence of risk factors are critical for Tanzania to achieve the health Sustainable Development Goals (SDG) for child mortality by 2030 [4].

This paper provides an account of the trends in the burden of diarrhea mortality in Tanzania from 1980 to 2015. The analysis takes into account changes in policy and coverage of diarrhea specific interventions and other sectoral interventions. We used the Lives Saved Tool (*LiST*) to assess the attribution of lives saved during the 1980-2015 to changes in coverage of interventions or in risk factors and projected the number of lives saved in 2030 for key diarrhea, nutrition and water, sanitation and hygiene (WASH) interventions.

## METHODS

We used a mixed method approach to analyze trends in diarrhea morbidity and mortality for this paper. To understand the contribution of the different policies, budgets and spending related to child diarrhea morbidity and mortality, we conducted a document review for policies, guidelines, grey literature, reports and policy briefs that we were able to find on the internet and from contacts at the Ministry of Health. We reviewed key government vision and policy documents including strategies for poverty reduction ie, National Strategy for Growth and Reduction of Poverty (NSGRP II) or MKUKUTA [5], Poverty Human Development Reports [6,7], Household Budget Surveys [8,9]. We reviewed government’s budget and spending on health using National Health Accounts (NHA) and Public Expenditure Reviews (PER) reports.

We reviewed National Health Policies, Health Sector Strategic Plans, and the national Sharpened One Plan to understand how programmatic and strategic changes in the sector affected implementation of child health programs including diarrhea. We consulted experts who were involved in the initial development and implementation of the control of diarrhea disease (CDD) program and IMCI strategy. We also interviewed other individuals working in maternal and child health programs in Tanzania, at the World Health Organization (WHO) country office and at Muhimbili University of Health and Allied Sciences (MUHAS) to get their account of how programs were implemented.

We assessed trends in coverage of child-health interventions related to diarrhea such as changes in stunting prevalence, ORS coverage, changes in wasting prevalence, changes in appropriate breastfeeding, improved sanitation, hand washing, vitamin A supplementation, hygienic disposal of child’s stools, improved water source, early initiation of breastfeeding, zinc for treatment of diarrhea, antibiotics for treatment of dysentery and coverage of rotavirus vaccine using all Tanzania Demographic and Health Surveys and Malaria Indicators Surveys 1991-92, 1999, 2004-05, 2010 and 2015-16. Coverage estimates of interventions prior to 1991 assumed 1980 coverage equal to earliest measured from nationally representative surveys. Estimates for subsequent years between surveys were done by linear interpolation between survey data points.

We also reviewed scientific literature and conducted systematic search of published literature on diarrhea mortality from Tanzania using key words. Other sectors such as nutrition, water and sanitation were also reviewed for information that was relevant to diarrhea mortality.

We used estimates of under-five mortality and diarrhea-specific mortality from the UN Inter-Agency Group for Mortality Estimation (IGME) and World Health Organization Maternal and Child Epidemiology Estimation (MCEE) group. The Lives Save Tool (*LiST* Version 5.62 Beta 39) was also used to assess the attribution in the reduction of diarrhea mortality from 1980 to 2015 to changes in coverage of the interventions and risk factors. We stratified results into pre-MDG (1980-2000) and post MDG era (2000-2015) in order to assess progress for the two periods. The general approach used in *LiST* is that interventions have an estimated efficacy in reducing cause-specific mortality or levels of risk factors. As intervention coverage increases, cause-specific mortality will decrease based on the magnitude of coverage change and the efficacy of the intervention [10,11].

## RESULTS

### Health reforms in Tanzania

Tanzania has gone through a series of health reforms since it got its independence in 1961. The Arusha Declaration in 1967 was a turning point for Tanzania as it adopted a policy of socialism and self-reliance [12]. Emphasis was put on social sectors including free education, health and access to clean water. Recession in the 1970s and 1980s forced Tanzania to implement a number of economic recovery and adjustment programs including the National Economic Survival Plan (NESP) 1980-1982, Structural Adjustment Programme (SAP), Economic Recovery Programme (ERP) I (1986) and II (1989); the Economic and Social Action Plan (ESAP) and Priority Social Action Plan (PSAP) (1989) [13]. These programs deliberately imposed cuts on allocation to social sectors which resulted in the decline of the quality of health service provision. Mortality rates stagnated and even started to increase, and morbidity rates rose.

In the early 1990s, Tanzania instituted major health sector reforms that affected the management, financing and organization of the health services [14]. User fees were introduced to complement constrained resources allocated in the health budget, health services were also decentralized and participation of the private sector was allowed. The sector wide approach (SWAp) was adopted for budget support. The percent expenditure of the total budget on health increased from 6% in 1980 to more than double in 2010/2011 [15].

### Diarrhea related policies and programmatic changes

**Figure 1:**
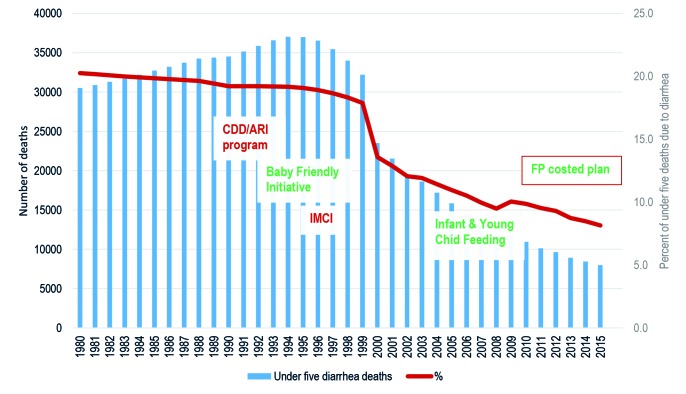
Diarrhea mortality trends, health sector policies and programs, 1980-2015.

The decline of diarrhea deaths can be associated with key policies and diarrhea-specific programs in Tanzania as shown in **Figure 1**. The National Control of Diarrheal Disease (CDD) program in Tanzania was established in early 1984 under the Epidemiology section of the Ministry of Health. The priority of the program was to reduce diarrheal related deaths and morbidity in children under five years through prevention and effective case management using oral rehydration therapy (ORT) and proper feeding. Emphasis was also put in increasing access in the number of facilities offering ORS, increasing quality of care for children seen at the facilities and instructing mothers on home-made solutions, continued feeding during diarrhea and diarrheal preventive measures. Diarrhea Treatment Corners (DTCs) were established in primary health care facilities where the preparation and use of Oral Rehydration Therapy (ORT) using Oral Rehydration Salt (ORS) and Sugar Salt Solution (SSS) was emphasized. Prevention activities of diarrhea were further extended to village level using scouts. Scouts went house to house training mothers how to prepare ORS and SSS. Treatment guidelines were later revised to allow community health workers to prepare and administer ORS in the village community health posts.

In 1990 the CDD program was merged with the acute respiratory infection (ARI) control program. In 1995, IMCI was introduced and a unit at the national level was established to coordinate all IMCI activities. This replaced the CDD/ARI program that was responsible for supporting child health interventions and diarrhea management became an integral part of IMCI. By 1998, IMCI was adopted as a national clinical approach. The Tanzania Essential Health Interventions Project (TEHIP) [16] from 1996-2003 research facilitated in building the momentum for evidence based planning in Tanzania, specifically for IMCI [17,18].

Baby Friendly Hospital Initiative was also launched in Tanzania in 1991 to promote and support breastfeeding. The initiative has not fared well because of lack of attention. Tanzania adapted the Global Strategy on Infant and Young Child Feeding and developed the National Strategy on Infant and Young Child Nutrition (IYCN) in 2005. Tanzania is also one of the 15 countries participating in the Global Action Plan for Preventable Pneumonia and Diarrhea (GAPPD) program [19,20]. Despite having high mortality, it has achieved most GAPPD targets and received one of the highest GAPPD score.

### Immunization program

Tanzania has had a well performing immunization program since its establishment in1975. Coverage of most antigens has been well over 80% since 2001 [21-23]. Although some fluctuation in performance was observed between 2004 and 2007, coverage of measles vaccine for children 12-23 months was 81% in 1990 and this has increased modestly to 86% in 2015. The percent of children 12-23 months receiving the first and second dose of rotavirus vaccine in 2015 was 94% and 89%, respectively [24].

Vitamin A supplementation (VAS), another key intervention was introduced in Tanzania in 1997 through the Essential Drug Program (EDP) and was given together with measles vaccine at 9 months. Bi-annual VAS campaigns for children 6-59 months were introduced in 2001 [25] increased coverage from less than 20% to nearly 90% in 2002 [26-28].

Despite quadrupling of funding for water and sanitation since 2002, Tanzania was not able to meet the Millennium Development Goal (MDG) for sanitation provision; 62% of population with access to improved sanitation by 2015 [29]. Population growth and rapid urbanisation are contributing factors in failure to meet this MDG target [30].

### Change in coverage/prevalence of interventions and risk factors

Data on coverage of interventions or prevalence of risk factors for diarrhea used in the *LiST* model are presented in **Table 1**. Over half of the children under 5 years attending health facilities in the year 2000 were reported to have received ORS for the treatment of diarrhea. This, however, declined to 45% in 2015. Less than one in five (18%) children under five years attending health facilities received zinc for the treatment of diarrhea. One third of children received treatment for persistent diarrhea in 2015. Coverage of rotavirus vaccine in 2015 was 98%.

**Table 1:** Coverage data for different diarrhea related factors or interventions in the year 1980, 2000 and 2015 used in the LiST analysis

Factors or interventions	1980 coverage (%)	2000 coverage (%)	2015 coverage (%)
Antibiotics for treatment of dysentery	0.0	0.0	0.0
Early initiation of breastfeeding	57.5	57.5	51.3
Hand washing with soap	4.0	10.2	13
Improved sanitation + improved water source	8.2	9.3	15.6
Rotavirus vaccine: two doses	0	0	98
ORS – oral rehydration salt solution	0.0	54.7	44.8
Persistent diarrhea treatment	0.0	0.0	33.0
Vitamin A supplementation	0.0	11.0	88.0
Zinc for treatment of diarrhea	0	0	17.5
Global stunting (<-2 SD) rate	44.9	48.6	34.2
Global wasting (<-2 SD) rate	8.0	4.8	4.5
Exclusive breastfeeding <1 month	50.5	58.4	86.8
No breastfeeding <1 month	1.5	0.7	0.2
Exclusive breastfeeding 1-5 months	25.3	30.7	58.7
Predominant breastfeeding 1-5 months	23.4	53.6	13.2
Partial breastfeeding 1-5 months	49.7	13.2	26.2
No breastfeeding 1-5 months	1.6	2.6	1.9
Any breastfeeding 6-11 months	97.8	96.7	98
Any breastfeeding 12-24 months	81	78.7	75.5

SD – standard deviation

Vitamin A supplementation coverage in children 6-59 months increased 8-fold from 11% in 2000 to 88% in 2015. Exclusive breastfeeding in children 1-5 months more than doubled from 25% 1980 to 59% in 2015. Similarly, exclusive breastfeeding for children less than one month of age increased from 51% to 87%. Early initiation of breastfeeding, however, declined from 56% in 1980 to 51% in 2015. Stunting was reduced by 10 percentage points from 45% in 1980 to 34% in 2015. Wasting was almost halved from 8% in 1980 to 5% during the same period.

All of the WASH indicators improved during the period. Handwashing tripled from 4% in 1980 to 13% in 2015, similarly, coverage of households with improved sanitation and improved water source increased from 8% to 16% in 1980 to 2015, respectively.

### Trends in under-five diarrhea mortality

**Figure 2:**
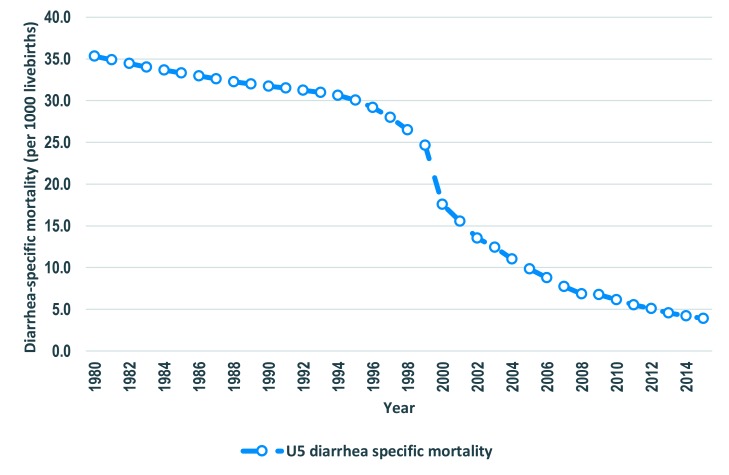
Annual rate of reduction of under-five diarrhea mortality in Tanzania, 1980-2015.

**Figure 3:**
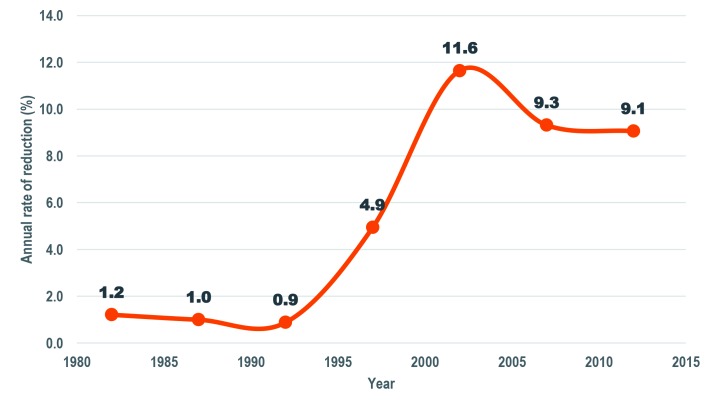
Diarrhea specific mortality among under-fives in Tanzania 1980-2015.

The number of diarrhea-specific deaths among under-five children declined by 74% from 30 521 in 1980 to 8000 in 2015 (**Figure 1**). Similarly, diarrhea-specific mortality rate (DSMR) among children under five years of age went down by 89% from 35.3 deaths per 1000 live births to 3.9 deaths per 1000 live births in the same period (**Figure 2**). The percent of under-five deaths due to diarrhea was reduced by more than one half from 21% in 1980 to 8% in 2015 (**Figure 1**). The annual rate of reduction (ARR) in diarrhea-specific mortality in under-fives was slightly above one in 1980 and remained more or less than same in the following decade. The ARR increased steadily to 4.9 in 1995-2000 and peaked at 11.6 in 2000-2005 before declining to 9.1 in 2010-2015 (**Figure 3**).

### *LiST* attribution of change in diarrhea mortality

**Table 2** shows the number of lives saved and the attribution of reduction in under five mortality to changes in intervention coverage and prevalence of risk factors. Major factors associated with diarrhea specific under five mortality reduction (DSMR) in 2000 compared to 1980 baseline were ORS (64%), changes in wasting prevalence (17%) and changes in age-appropriate breastfeeding practices (12%) accounting for 25 071 (93%) of the number of lives saved. In 2015 compared to 2000 baseline, changes in stunting prevalence was associated with one third of the DSMR reduction: vitamin A supplementation accounted for a quarter and rotavirus vaccine one fifth of the reduction.

**Table 2:** Lives saved and the percent of the diarrhea specific under-five mortality reduction attributable to each factor in Tanzania for the periods 1980-2015, 1980-2000 and 2000-2015

	2000 compared to 1980		2015 compared to 2000		2015 compared to 1980	
**Intervention**	**Lives saved**	**Reduction attributable (%)**	**Lives saved**	**Reduction attributable (%)**	**Lives saved**	**Reduction attributable (%)**
Zinc for treatment of diarrhea	0	0.00	1071	5.5	1480	2.9
Vitamin A supplementation	838	3.11	4812	24.5	8205	16.2
Rotavirus vaccine	0	0.00	4103	20.9	6506	12.9
ORS – oral rehydration salt solution	17308	64.15		0.0	12252	24.2
Improved water + sanitation	168	0.62	723	3.7	1378	2.7
Early initiation of breastfeeding	40	0.15		0.0	6	0.0
Changes in age-appropriate breastfeeding practices	3223	11.95	178	0.9	3840	7.6
Changes in wasting prevalence	4540	16.83	466	2.4	4865	9.6
Changes in stunting prevalence	0	0.00	6402	32.7	8355	16.5
Antibiotics for dysentery	0	0.00		0.0		0.0
Hand washing with soap	864	3.20	300	1.5	1547	3.1
Persistent diarrhea treatment	0	0.00	1550	7.9	2141	4.2
Total	26981	100.0	19605	100.0	50575	100.0

Over fifty thousand lives were saved in 2015 compared to 1980 baseline where the main factors associated with DSMR reduction in under-fives were ORS (24%), changes in stunting prevalence (17%), vitamin A supplementation (16%), rotavirus vaccine (13%), changes in wasting prevalence (10%) and changes in age-appropriate breastfeeding practices (8%). Table S1 in **Online Supplementary Document** provide these estimates with upper and lower bounds.

### *LiST* projections of possible diarrhea mortality decline by 2030

The projected number of lives saved and the DSMR in under-fives that is attributed to changes in intervention coverage and prevalence of risk factors for three scale-up scenarios is presented in **Table 3**. Table S2 in **Online Supplementary Document** provides these estimates with upper and lower bounds. Scenario 1 shows the impact of scaling up five diarrhea specific interventions ie, ORS, zinc, persistent diarrhea treatment, antibiotics for dysentery and rotavirus vaccine. Overall, scaling coverage of these five interventions would reduce DSMR by almost 75% (from 4.5 to 1.1) (**Table 4**). Scale up of ORS alone accounted for more than half of the lives saved in this scenario.

**Table 3:** Projected number of lives saved and the percent reduction in diarrhea specific under-five mortality attributable to scaling up different packages of intervention for the three different scenarios by 2030

Factors/Intervention	Direct diarrhea interventions (Scenario 1)		Direct diarrhea interventions and nutrition (Scenario 2)		Direct diarrhea interventions, nutrition and WASH (Scenario 3)	
**No. of lives saved**	**Reduction attributable (%)**	**No. of lives saved**	**Reduction attributable (%)**	**No. of lives saved**	**Reduction attributable (%)**
Zinc for treatment of diarrhea	1557	16.7	1004	9.6	610	5.4
Vitamin A supplementation	0	0.0	75	0.7	61	0.5
Rotavirus vaccine	382	4.10	354	3.4	283	2.5
ORS	5356	57.5	3500	33.3	2125	18.8
Improved sanitation	0	0.0		0.0	2070	18.3
Early initiation of breastfeeding	0	0.0	6	0.1	5	0.04
Changes in age-appropriate breastfeeding practices	0	0.0	1587	15.1	1247	11.1
Changes in wasting prevalence	0	0.0	95	0.9	58	0.5
Changes in stunting prevalence	0	0.0	2577	24.5	2060	18.3
Antibiotics for dysentery	807	8.7	518	4.9	315	2.8
Hand washing with soap	0	0.0	0	0.0	1973	17.5
Persistent Diarrhea Treatment	1221	13.1	787	7.5	478	4.2
Total	9323	100.0	10503	100.0	11285	100.0

ORS – oral rehydration solution, WASH – water, sanitation and hygiene

**Table 4:** The impact on diarrhea-specific mortality (DSMR) if universal coverage of different packages of interventions is achieved by 2030.

2015	2030					
	**Scenario 1**		**Scenario 2**		**Scenario 3**	
**DSMR**	**DSMR**	**Percent Reduction**	**DSMR**	**Percent Reduction**	**DSMR**	**Percent Reduction**
4.5	1.1	74.5%	0.7	84.0%	0.4	90.3%

Scenario 2 shows the impact of scaling up nutrition interventions along with the direct diarrhea interventions. In this scenario, DSMR would drop 0.7 (**Table 4**). Changes in stunting prevalence and in age-appropriate breastfeeding practices were the main factors associated with an additional lives saved when added to direct diarrhea interventions.

Scenario 3, adds the impact of scaling up WASH interventions along with the direct diarrhea and nutrition interventions. In this scenario DSMR drops 90% to 0.4 (**Table 4**). Changes in coverage of handwashing with soap and improved sanitation was associated with a 18% and 18% respectively of lives saved when direct diarrhea, nutrition and WASH interventions were scaled up, while as in the other scenarios scale up of ORS still is accountable for the largest reduction in diarrhea mortality (19%). Table S3 in **Online Supplementary Document** provides these estimates with upper and lower bounds.

## DISCUSSION

Tanzania has made significant progress in reducing diarrhea specific mortality in children under five years old between 1980 and 2015. This progress is associated with a number of factors, mainly high coverage of EPI vaccines including rotavirus, vitamin A supplementation and the integration of child health services notably the CDD program and IMCI. The average annual rate of reduction (ARR) in diarrhea specific under-five mortality was low from 1980 to the mid-1990s, but increased remarkably in the 2000s. The rapid increase in ARR in the 2000s is probably associated with optimal implementation of IMCI in the early years from 1998-2010. This included the role of nutrition, pharmacists, and health care workers, but foremost, it empowered care-takers to take charge and recognize danger signs.

In 2012, Tanzania became the eleventh GAVI-eligible country to introduce rotavirus vaccine in the national immunization program. Coverage of two doses of rotavirus vaccine in 2015 were 94% and 89% respectively. The immunization program in Tanzania has traditionally performed well. Coverage of almost all antigens has been over 80% since the establishment of the EPI program. The high coverage of rotavirus vaccine within a short time of its introduction is evidence of strong leadership and the service platform in the program.

Prevention and effective management of diarrhea with ORS was one of the key tenets of the National Control of Diarrheal Disease (CDD) for the reduction of diarrhea related morbidity and mortality. Up to 2005, Tanzania was one of the few countries in sub-Saharan Africa with the highest coverage of ORS [31]. ORS accounted for more lives saved as a single intervention in the year 2000 with a 1980 baseline and in 2015 with a 1980 baseline. Coverage of ORS however, has declined in the recent past [24,32]. This has been coupled with change in priorities at the district level as well as change in staff who were trained in IMCI who either have retired or moved out of the health system. Although zinc was recommended for the treatment of diarrhea in under-fives, and campaigns by the Ministry of Health were conducted to promote use, demand of zinc by caregivers and uptake was low [33]. This was partly due to IMCI training rollout that did not include the use of zinc for the management of diarrheal diseases. The introduction of accredited drug dispensing outlets (ADDOs) has also increased access of quality health services and commodities including zinc and antimalarial for treatment of diarrhea and malaria [34] respectively.

Health sector reforms have been instrumental in expanding access to health services in Tanzania. Soon after independence in 1961, Tanzania adopted a free health services policy which lasted until the 1990s when user fees were reintroduced. Access to health care for the poor was through exemptions and waivers. The community health fund (CHF) established in 2001 is another mechanism that was meant to increase health services access for rural communities who are mostly in the informal sector. Coverage of CHF is however, still below 10% of the total population and the national health insurance fund still covers only 15% of the total population. Efforts are under way to establish a single national health insurance scheme that is likely to pull more resources and increase coverage to the poor and vulnerable population. The newly introduced direct health facility financing (DHFF) in 2017 is another mechanism that will bolster health services delivery particularly at the lower levels [35].

Projections of universal coverage of diarrhea interventions and the reduction of associated risk factors using the *LiST* model, show the potential of reducing DSMR in under-fives to negligible levels by 2030. These projections also provide guidance on where a country like Tanzania should focus its investments in the next few years if we aim to attain the SDG goals in 2030 of leaving no one behind. Universal coverage of ORS, zinc, rotavirus vaccine and treatment of persistent diarrhea by 2030 is attributed to 80% reduction in DSMR. The reduction in stunting prevalence and the addition of nutritional interventions would result in important reductions (40%) in DSMR. The impact of near universal coverage on DSMR if universal coverage of diarrhea, nutrition and WASH intervention are implemented by 2030 is substantial.

Tanzania has been fortunate to have strong leadership at the forefront of the child survival era that committed resources and spearheaded the gains observed. Smooth transitioning from vertical programs for child health such as CDD to integrated programs like CDD/ARI and later IMCI were important in keeping the momentum in addressing major causes of child deaths including diarrhea. Projections from *LiST* show that scaling up diarrhea, nutrition and WASH interventions have the potential to significantly reduce DSMR. We believe that strengthening of the community health workers program to participate in the delivery of child health interventions and to increase financial protection through health insurance cover for the poor and vulnerable groups, will be critical in the next few years for Tanzania to reach the health SDG targets in 2030.

## CONCLUSION

Overall, there has been progress in diarrhea specific under five mortality reduction (DSMR) in Tanzania from 1980 to 2015. The health sector reforms instituted after independence contributed to the gains we have documented. These however, were challenged with structural adjustment programs. We believe SDG targets are achievable in Tanzania if policies that target the poor in terms of increasing access to health, education, clean and safe water are key in addressing childhood diarrhea disease and deaths. The current expansion of health facilities to lower levels should serve the increased population that has been observed in the last decade with the availability of equipment, supplies and trained personnel for case management. While developments are welcome, it is important to ensure integration of key childhood interventions are integrated in order to achieve maximum impact.

## Additional material

Online Supplementary Document
