# Supplementary material for: Factors associated with the decline in under five diarrhea mortality in Tanzania from 1980-2015
Source: J Glob Health. 2019 Oct 22;9(2):020806. doi: 10.7189/jogh.09.020806 (PMC6816318; doi:10.7189/jogh.09.020806)
Supplement: Online Supplementary Document [file jogh-09-020806-s001.pdf]

Ranges around estimates

To develop ranges around our estimates of lives saved and lives saved by interventions we varied the efficacy of the interventions within the LiST Saved Tool as well as the risk ratios that link stunting and wasting to mortality. The ranges are based up the 95% confidence bounds around the estimates of efficacy and risk for interventions where meta-analyses have been performed. For some interventions, primarily around birth care, estimates of efficacy are based on a Delphi procedure and for these values we used inter-quartile ranges as the bounds for efficacy. The Lives Saved Tool contains the source of estimates of intervention efficacy as well as the risk ratios used in the model. More details on these values can also be found at the Lives Saved Tool website, www.livessavedtool.org.

Below we present the three tables showing estimates of lives saved and diarrhea specific mortality rates from the main text with upper and lower bounds for each estimate.

Table S1. Lives saved and the percent of the diarrhea specific under-five mortality reduction attributable to each factor in Tanzania for the periods 1980-2015, 1980-2000 and 2000-2015. Ranges around the estimates are in parentheses.

	2000 compared to 1980		2015 compared to 2000		2015 compared to 1980	
**Intervention**	**Lives Saved**	**Reduction attributable (%)**	**Lives Saved**	**Reduction attributable (%)**	**Lives Saved**	**Reduction attributable (%)**
Zinc for treatment of diarrhea	0	0.0	1071 (820,1267)	5.5 (4.2, 6.5)	1480 (1203,1716)	2.9 (2.9,3.1)
Vitamin A supplementation	838 (561,1063)	3.1 (2.1, 3.9)	4812 (3258, 6046)	24.5 (16.3, 30.1)	8205 (5748,10079)	16.2 (13.6,18.2)
Rotavirus vaccine	0	0.0	4103 (1969, 5456)	20.9 (10.0, 27.8)	6506 (3135, 8570)	12.9 (7.4, 15.5)
ORS	17308 (15939,18463)	64.1 (59.0, 68.4)	0	0.0	12252 (11343, 13534)	24.2 (20.5, 32.1)
Improved water + sanitation	168 (108, 231)	0.6 (0.4, 0.8)	723 (487, 968)	3.7 (2.5, 5.0)	1378 (936, 1825)	2.7 (2.2 ,3.3)
Early initiation of breastfeeding	40 (39, 40)	0.1 (0.1,0.1)	0	0.0	6 (5, 6)	0.0
Changes in age-appropriate breastfeeding practices	3223 (2698, 3831)	11.9 (10.0, 14.1)	178 (0,1078)	0.9 (0.0, 5.5)	3840 (2307, 4286)	7.6 (5.5, 8.1)
Changes in wasting prevalence	4540 (3763, 5384)	16.8 (13.9,19.9)	466 (337, 482)	2.4 (1.7, 2.5)	4865 (4618, 5045)	9.6 (9.1, 10.9)
Changes in stunting prevalence	0	0.0	6402 (5464, 7208)	32.7 (27.9, 36.8)	8355 (7015, 9446)	16.5 (16.6 ,17.1)
Antibiotics for dysentery	0	0.0	0	0.0	0	0.0
Hand washing with soap	864 (538, 1202)	3.2 (2.0, 4.4)	300 (194, 411)	1.5 (1.0, 2.1)	1547 (1014, 2079)	3.1 (2.4 ,3.8)
Persistent diarrhea treatment	0	0.0	1550 (1360, 1820)	7.9 (6.9, 9.3)	2141 (1842, 2669)	4.2 (6.3, 3.3)
Total	26981 (23647,28813)		19605 (14494, 24131)		50575 (42185 ,55336)	

Table S2. Projected number of lives saved and the percent reduction in diarrhea specific under-five mortality attributable to scaling up different packages of intervention for the three different scenarios by 2030. Ranges around the estimates are in parentheses.

Factors/Intervention	Direct diarrhea interventions (Scenario 1)		Direct diarrhea interventions and nutrition (Scenario 2)		Direct diarrhea interventions, nutrition and WASH (Scenario 3)	
	No. of lives saved	Reduction attributable (%)	No. of lives saved	Reduction attributable (%)	No. of lives saved	Reduction attributable (%)
Zinc for treatment of diarrhea	1557 (1079, 1987)	16.7 (13.4, 19.4)	1004 (848, 1072)	9.6 (9.4, 9.6)	610 (519, 627)	5.4 (4.6, 5.6)
Vitamin A supplementation	0	0.0	75 (42, 115)	0.7 (0.5, 1.0)	61 (36 ,86)	0.5 (0.3, 0.8)
Rotavirus vaccine	382 (171, 524)	4.1 (2.1, 5.1)	354 (165, 472)	3.4 (1.8, 4.2)	283 (142, 352)	2.5 (1.3, 3.1)
ORS	5356 (4792, 5664)	57.5 (59.4, 55.4)	3500 (3112, 3777)	33.3 (27.5, 41.8)	2125 (1507, 2795)	18.8 (13.4, 24.8)
Improved sanitation	0	0.0	0	0.0	2071 (1520, 2520)	18.4 (13.5, 22.3)
Early initiation of breastfeeding	0	0.0	6 (6, 6)	0.1 (0.1, 0.1)	5 (5, 5)	0.0 (0.0, 0.1)
Changes in age-appropriate breastfeeding practices	0	0.0	1587 (448, 2399)	15.1 (5.0, 21.2)	1247 (387, 1734)	11.0 (3.5, 15.4)
Changes in wasting prevalence	0	0.0	95 (88,97)	0.9 (1.0,0.9)	58 (47, 65)	0.5 (0.4, 0.6)
Changes in stunting prevalence	0	0.0	2577 (2072, 2961)	24.5 (22.9, 26.1)	2060 (1781, 2193)	18.3 (15.8, 19.4)
Antibiotics for dysentery	807 (711, 910)	8.7 (8.8, 8.9)	518 (488, 559)	4.9 (6.2, 4.3)	315 (236, 413)	2.8 (2.1, 3.7)
Hand washing with soap	0	0.0	0	0.0	1973 (1395, 2440)	17.5 (12.4, 21.6)
Persistent Diarrhea Treatment	1220 (1139, 1316)	13.1 (11.1, 16.3)	787 (614, 1035)	7.5 (11.4, 5.4)	478 (297, 766)	4.2 (2.6, 6.8)
Total	9322 (8069, 10224)		10503 (9040, 11336)		11286 (7883, 13996)	

Table S3. The impact on diarrhea-specific mortality (DSMR) if universal coverage of different packages of interventions is achieved by 2030. Ranges around the estimates are in parentheses.

2015	2030					
	Scenario 1		Scenario 2		Scenario 3	
DSMR	DSMR	Percent Reduction	DSMR	Percent Reduction	DSMR	Percent Reduction
4.5	1.1 (0.8,1.1)	74.5% (64.5%,81.8%)	0.7 (0.4,1.2)	84.0% (72.2%,90.6%)	0.4 (0.2,0.9)	90.3% (79.5%,94.4%)
